# Unveiling Variations: A Comprehensive Comparison of Five Globally Used Thyroid Cytology Reporting Systems With Histopathological Correlation

**DOI:** 10.7759/cureus.53359

**Published:** 2024-02-01

**Authors:** Sudhakar Ramamoorthy, Amulya Boddapati, Inuganti Venkata Renuka, Santhi Imandi, Bellamkonda Mounica

**Affiliations:** 1 Pathology, NRI Medical College, Chinakakani, IND

**Keywords:** bethesda, cytology reporting systems, fine needle aspiration cytology (fnac), thyroid cytology, thyroid neoplasms

## Abstract

Introduction

Accurate cytological assessment is pivotal for managing thyroid lesions and various global reporting systems are in use, such as the globally acclaimed The Bethesda System for Reporting Thyroid Cytopathology (TBSRTC), alongside other reporting systems namely, the Japanese Reporting System for Thyroid Aspiration Cytology (JRSTAC), Italian Consensus for the Classification and Reporting of Thyroid Cytology (ICCRTC), the UK Royal College of Pathologists System for Reporting Thyroid Cytopathology (UK RCPath), the Royal College of Pathologists of Australasia and Australian Society of Cytology Classification System (RCPA/ASC). Notably, variations exist among these systems which are influenced by country-specific statistics. Given the lack of large-scale data in India and the difference in prevalence of diagnostic entities compared to the western population, this study aimed to identify reporting systems suitable for the Indian population focusing on distinguishing neoplastic from non-neoplastic lesions.

Materials and methods

A cross-sectional analysis of 40 thyroid cytology cases with histopathological correlation was conducted. Pathologists independently assessed cytology slides using JRSTAC, ICCRTC, RCPA/ASC, UK RCPath and TBSRTC. Five performance indicators, sensitivity, specificity, positive predictive value (PPV) of neoplastic conditions, negative predictive value (NPV) of non-neoplastic conditions, diagnostic accuracy and two quality indicators, percentage of Atypia of undetermined significance (AUS) and AUS/Malignant ratio were analyzed and compared.

Results

Among 40 cases, 22 cases were neoplastic (16 papillary thyroid carcinoma, six follicular adenoma) and 18 non-neoplastic (14 multinodular goiter, four lymphocytic thyroiditis). Specific patterns emerged in cases labeled “Non-diagnostic”, prompted questions about categorizing inadequately cellular cases as “benign” in light of the presence of specific findings. All reporting systems showed 100% specificity in detecting non-neoplastic and neoplastic conditions in Category 1 and Category 6 respectively. Performance and quality indicators varied among reporting systems with TBSRTC (PPV of neoplastic cases 85.71%, NPV of non-neoplastic cases 70.58%, specificity 85.7%, sensitivity 70.58%, diagnostic accuracy 60%, AUS percentage 22.5% and AUS/Malignant ratio 3%) and RCPA/ASC (PPV of neoplastic cases 76.47%, NPV of non-neoplastic cases 70.58%, specificity 75%, sensitivity 72.2%, diagnostic accuracy 62.5%, AUS percentage 15% and AUS/Malignant ratio 3%) showing better results.

Conclusion

Among the five thyroid cytology reporting systems studied, TBSRTC and RCPA/ASC showed better overall performance results and quality indicators were close to benchmark. Better performance by TBSRTC 2023 could be due to the detailed criterion mentioned per category with subcategorization of AUS and suspicious for malignancy by features of cytological and architectural atypia. Similarly, RCPA/ASC has subcategorized AUS with defined criteria and certain background features were included as an isolated criterion for the suspicious for malignancy category. These defined criteria outlined in TBSRTC and RCPA/ASC played a crucial role in minimizing and reclassifying cases from the indeterminate categories (AUS and suspicious for malignancy) into well-defined categories with established management protocols.

## Introduction

Thyroid lesions are frequently encountered in clinical practice, and the accuracy of cytological assessment plays a pivotal role in guiding appropriate patient management. It has been estimated that over 50% of the general population [1], although asymptomatic, possess radiologically detectable thyroid nodules in which only a marginal 5% exhibit malignancy [2]. Thyroid neoplasms are one of the most common neoplasms that show high inter-observer and intra-observer variability in cytology [3,4]. Hence to ensure high precision and to facilitate risk stratification, numerous reporting systems have been devised for categorizing thyroid cytology specimens into diagnostic classes.

In 1996, the Papanicolaou Society of Cytopathology Task Force on Standards of Practice took the initiative to standardize thyroid cytology reporting [5]. Subsequent to this, the Japanese Reporting System for Thyroid Aspiration Cytology (JRSTAC) was introduced in 2005, offering a more structured framework, which was subsequently refined into the currently adhered JRSTAC 2019 reporting system [6]. This evolution has been mirrored globally with different countries adopting their own reporting guidelines, including the Italian Consensus for the Classification and Reporting of Thyroid Cytology (ICCRTC 2016) [7], the UK Royal College of Pathologists System for Reporting Thyroid Cytopathology (UK RCPath 2016) [8], the Royal College of Pathologists of Australasia and Australian Society of Cytology (RCPA/ASC 2019) classification system [9], and The Bethesda System for Reporting Thyroid Cytopathology (TBSRTC 2023) [10].

Non-diagnostic, benign, and malignant categories

Across the reporting systems, diagnostic criteria for the malignant, benign, and adequacy (at least six groups with 10 cells in each group preferably per slide) categories remain consistent. However, each system has different sets of exceptions for adequacy. Architectural or cytological atypia commonly fall within adequacy exceptions. Notably, a prominent lymphocytic infiltrate in a low cellular smear is considered adequate and benign across all systems, except RCPath. In cases lacking sufficient cellularity/inflammation/atypia, the presence of either a cyst or colloid is deemed sufficient to categorize the lesion as benign in JRSTAC, RCPA/ASC, and Bethesda systems. However, ICCRTC and RCPath stipulate the presence of colloid in cystic lesions to consider adequate. A distinct category is assigned to cystic lesions with inadequate cellularity in JRSTAC which are categorized as benign unless radiological suspicion of malignancy exists. This was due to Japanese population studies that demonstrated minimal malignancy (0.2%) in pure cystic lesions [11].

Atypia of undetermined significance, follicular neoplasm, and suspicious for malignancy

The term "atypia" encompasses both architectural and cytological atypia. Architectural atypia includes patterns such as papillae and microfollicles, while cytological atypia includes anisonucleosis, irregular nuclear membranes, chromatin texture, and prominent nucleoli [12]. The lack of standardized definitions contributes to interobserver variability in the atypia of undermined significance category, manifesting in divergent criteria among the reporting systems. Bethesda, RCPath, and RCPA/ASC reporting guidelines incorporate both architectural and cytological abnormalities as criteria for atypia. In contrast, JRSTAC solely considers cytological abnormalities as indicative of atypia. Consequently, the Japanese system identifies all thyroid neoplasms, excluding follicle-based neoplasms, in the absence of nuclear atypia. In contrast, ICCRTC limits atypia criteria to architectural anomalies in TIR3A, yielding a lower malignancy risk in comparison to its Bethesda counterpart [13,14].

Unlike other reporting systems, which categorize follicle-based lesions as AUS or follicular neoplasms based on supplementary cytological findings, JRSTAC designates all follicular lesions as belonging to the follicular neoplasm category, regardless of nuclear findings. This decision was made due to the low prevalence of NIFTP among the Japanese population, hence NIFTP's existence in the follicular neoplasm category exerts minimal influence on the risk of malignancy (ROM). This categorization also takes into consideration the distinct mutation pathway (RAS) associated with these follicular lesions (NIFTP, Follicular adenoma, Follicular carcinoma, Follicular variant of papillary thyroid carcinoma) [15]. In the suspicious for malignancy category, JRSTAC, ICCRTC, and UK RCPath systems adopt analogous criteria, defining cases as such when they exhibit mixed benign and malignant features or when the findings fall short of malignancy, both quantitatively and qualitatively. The RCPA/ASC system introduces background malignancy features (like psammoma bodies) as an isolated criterion, enhancing the sensitivity for detecting malignancy [13]. The most recent TBSRTC 2023 reporting system introduces numerous modifications in the intermediate category (AUS and Suspicious for malignancy). Where feasible, Bethesda subdivides these intermediate categories with precise explanations, some of which are quantifiable, to mitigate subjective variations, thus enhancing the diagnostic accuracy for both benign and malignant thyroid lesions.

## Materials and methods

**Figure 1:**
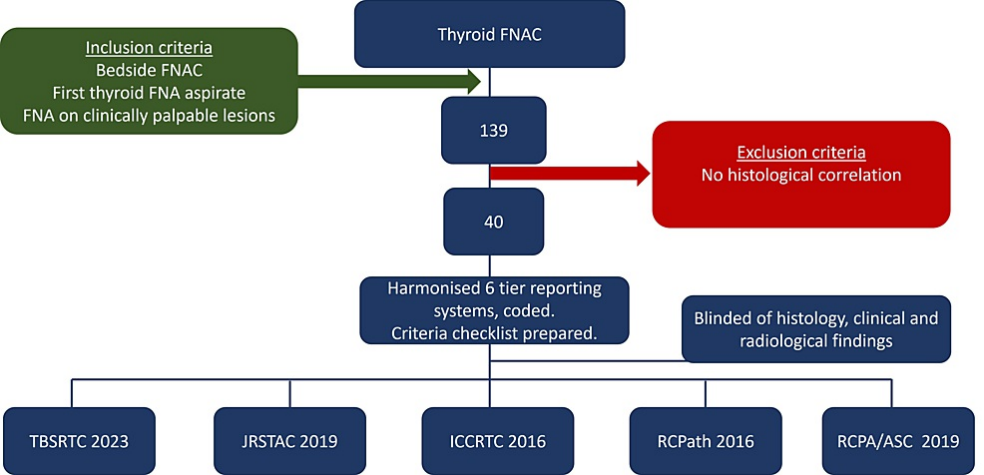
Methodology workflow of the current study TBSRTC 2023: The Bethesda System for Reporting Thyroid Cytopathology 2023; JRSTAC 2019: Japanese Reporting System for Thyroid Aspiration Cytology 2019; ICCRTC 2016: Italian Consensus for the Classification and Reporting of Thyroid Cytology 2016; UK RCPath 2016: The UK Royal College of Pathologists System for Reporting Thyroid Cytopathology 2016; RCPA/ASC 2019: The Royal College of Pathologists of Australasia and Australian Society of Cytology 2019; FNAC: Fine needle aspiration cytology

This study entails a cross-sectional analysis of 40 cases of thyroid cytology that has a histopathology correlation. Thyroid fine needle aspiration cytology cases reported in our institute in the past year were 139. Of these 139 cases, 40 cases had histological follow-up. Cytology slides of these 40 cases were retrieved from the archives. The data collection process involved independent examination of cytology slides of these 40 cases by five pathologists (each assigned to report using one reporting system) who were blinded to histopathology diagnosis, as well as the clinical and radiological information associated with each case. Figure 1 outlines the study methodology with inclusion criteria, exclusion criteria, case selection and cytology reporting systems.

Each case was meticulously classified according to the established reporting systems, including JRSTAC, ICCRTC, RCPA/ASC, UK RCPath, and TBSRTC. Table 1 delineates the categories of the five distinct thyroid cytology reporting systems that were used worldwide.

**Table 1:** Thyroid cytopathology reporting systems and their respective categories TBSRTC 2023: The Bethesda System for Reporting Thyroid Cytopathology 2023; JRSTAC 2019: Japanese Reporting System for Thyroid Aspiration Cytology 2019; ICCRTC 2016: Italian Consensus for the Classification and Reporting of Thyroid Cytology 2016; UK RCPath 2016: The UK Royal College of Pathologists System for Reporting Thyroid Cytopathology 2016; RCPA/ASC 2019: The Royal College of Pathologists of Australasia and Australian Society of Cytology 2019

TBSRTC 2023 [10]		JRSTAC 2019 [6]		ICCRTC 2016 [7]		UK RCPath 2016 [8]		RCPA/ASC 2019 [9]	
I	Non-diagnostic	I	Unsatisfactory	TIR1	Non-diagnostic	Thy1	Non-diagnostic	I	Non-diagnostic
		II	Cyst fluid	TIRc	Non-diagnostic -cystic	Thy1c	Non-diagnostic – cystic		
II	Benign	III	Benign	TIR2	Benign	Thy2 Thy2c	Non-neoplastic Non-neoplastic – cystic	II	Benign
III	Atypia of Undetermined Significance	IV	Undetermined significance	TIR3A	Low-risk intermediate lesion	Thy3a	Neoplasm possible, atypia	III	III Intermediate or follicular lesion of undetermined significance
IV	IVa Follicular neoplasm IVb Oncocytic follicular neoplasm	V	Follicular neoplasm	TIR3B	High-risk intermediate lesion	Thy3f	Neoplasm possible, follicular	IV	Suggestive of follicular neoplasm
V	Suspicious for malignancy	VI	Suspicious for malignancy	TIR4	Suspicious for malignancy	Thy4	Suspicious for malignancy	V	Suspicious for malignancy
VI	Malignant	VII	VII Malignant	TIR5	Malignant	Thy5	Malignant	VI	Malignant

Since different reporting protocols followed different tiered systems, for the ease of analysis and simplification, all the reporting systems used in the study were harmonized to 6-tier reporting systems, and unique codes were assigned. Importantly, this consolidation was executed without altering the diagnostic criteria stipulated in the original reporting systems for each category. The modified category nomenclature of the reporting systems, following the simplification, is detailed in Table 2.

**Table 2:** Modified category nomenclature used for analytical purpose of the study TBSRTC 2023: The Bethesda System for Reporting Thyroid Cytopathology 2023; JRSTAC 2019: Japanese Reporting System for Thyroid Aspiration Cytology 2019; ICCRTC 2016: Italian Consensus for the Classification and Reporting of Thyroid Cytology 2016; UK RCPath 2016: The UK Royal College of Pathologists System for Reporting Thyroid Cytopathology 2016; RCPA/ASC 2019: The Royal College of Pathologists of Australasia and Australian Society of Cytology 2019

TBSRTC 2023 [10]		JRSTAC 2019 [6]		ICCRTC 2016 [7]		UK RCPath 2016 [8]		RCPA/ASC 2019 [9]	
Category	Code	Category	Code	Category	Code	Category	Code	Category	Code
I	B1	I, II	J1	TIR1, TIR1c	I1	I	A1	Thy1, Thy1c	R1
II	B2	III	J2	TIR2	I2	II	A2	Thy2, Thy2c	R2
III	B3	IV	J3	TIR3A	I3	III	A3	Thy3a	R3
IV	B4	V	J4	TIR3B	I4	IV	A4	Thy3f	R4
V	B5	VI	J5	TIR4	I5	V	A5	Thy4	R5
VI	B6	VII	J6	TIR5	I6	VI	A6	Thy5	R6

Through this comprehensive approach, the study aims to provide an in-depth comparison of the diagnostic accuracy and other performances of these reporting systems in identifying neoplastic lesions.

## Results

In our study, 29 patients (29/40, 72.5%) were females and 11 patients (11/40, 27.5%) were males. The mean age at the time of diagnosis was found to be 39.55 +/- 14.7 years and the median age group was 40 years. Out of the total 40 thyroid cases meticulously examined in this study, 22 cases were unequivocally verified as neoplastic conditions through histopathology, while the remaining 18 cases were identified as non-neoplastic entities. It's worth noting that our study primarily focused on differentiating neoplastic from non-neoplastic conditions rather than the conventional benign versus malignant distinction. This approach was adopted to align with the primary purpose of thyroid cytology, which serves as a preliminary screening tool aimed at guiding clinicians toward surgical management decisions, rather than providing an absolute diagnostic outcome.

In our analysis, categories 4, 5, and 6 from the various reporting systems that typically signify the need for surgical consideration were collectively labeled as "Positive" outcomes. Within this "Positive" grouping, cases that were indeed confirmed as neoplastic through biopsy were classified as "true positives”. Conversely, categories 1 and 2 were designated as "Negative" for our analysis. Within this category grouping, cases which were verified as non-neoplastic through biopsy were classified as "True negatives”.

**Figure 2:**
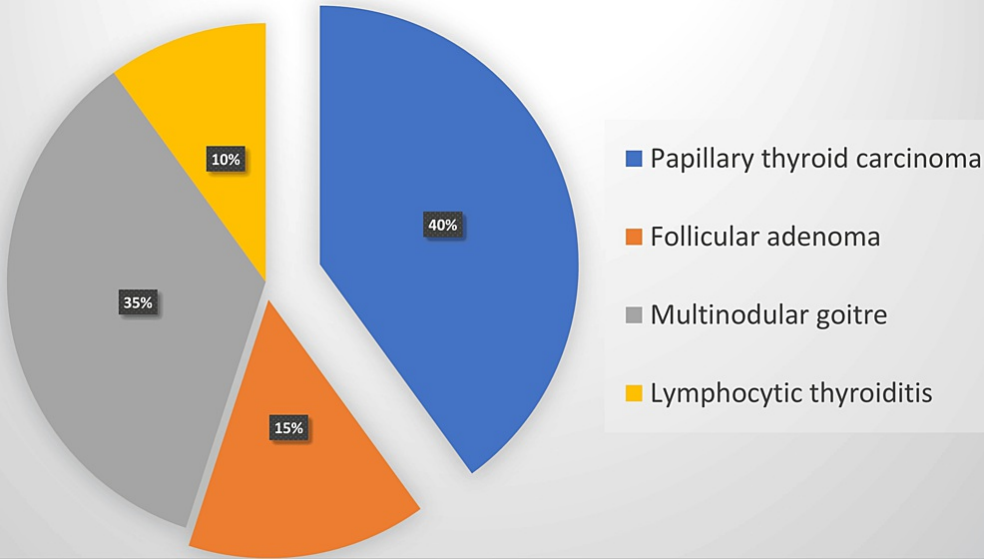
Frequency distribution of histopathology diagnoses in the current study

Our analysis revealed that among the neoplastic conditions, in histopathology 16 cases were identified as Papillary thyroid carcinoma, while the remaining six cases were attributed to follicular adenoma. On the other hand, within the non-neoplastic category, 14 cases were diagnosed as multinodular goiter, and an additional four cases were recognized as lymphocytic thyroiditis. Figure 2 provides a clear representation of the frequency distribution across these various conditions. Among the reporting systems, TBSRTC and RCPA/ASC achieved better results with sensitivity, specificity and p-value respectively were 70.58%, 85.7%, 0.008 and 72.2%, 75%, 0.03. The newly introduced quality indicators in TBSRTC 2023 were attempted in the current study and showed better results in RCPA/ASC and TBSRTC close to the benchmark. (AUS:<10%, AUS/Malignant ratio: <3) [10].

**Figure 3:**
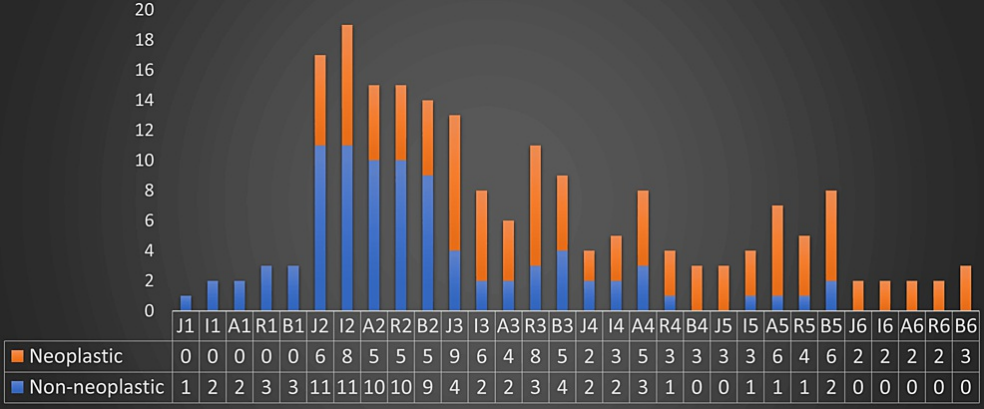
Clustered column chart depicting the distribution of cytology cases across categories in five reporting systems

The column chart in Figure 3 illustrates the frequency distribution of cytological diagnostic categories across all five reporting systems. Out of the 18 confirmed non-neoplastic cases through biopsy, an interesting pattern has emerged. Two cases were consistently labeled as "Non-diagnostic" in their cytology reports by all reporting systems except JRSTAC. This classification was attributed to limited cellularity accompanied by features like polymorphous lymphocytes, cystic fluid, or abundant colloid. Surprisingly, none of these cases were later diagnosed as malignant in histology. This raises a question: Should cases with inadequate cellularity be automatically categorized as "benign" if any of these specific features (polymorphous lymphocytes, cystic fluid, or abundant colloid) are present? This observation prompts a deeper exploration of cases with suboptimal cellularity and the implications of using these additional findings in inadequate cellularity to consider “benign”.

From Figure 3 it was evident that all reporting systems showed 100% specificity in identifying non-neoplastic and neoplastic lesions in Category 1 and Category 6 respectively.

Table 3 depicts the performance indicators of thyroid cytology reporting systems in diagnosing neoplastic conditions. Among the reporting systems, TBSRTC and RCPA/ASC achieved better results with sensitivity, specificity and p-value respectively were 70.58%, 85.7%, 0.008 and 72.2%, 75%, 0.03. The newly introduced quality indicators in TBSRTC 2023 were attempted in the current study and showed better results in RCPA/ASC and TBSRTC close to the benchmark. (AUS:<10%, AUS/Malignant ratio: <3) [10].

**Table 3:** Comparison of performance indicators of thyroid cytology reporting systems TBSRTC 2023: The Bethesda System for Reporting Thyroid Cytopathology 2023; JRSTAC 2019: Japanese Reporting System for Thyroid Aspiration Cytology 2019; ICCRTC 2016: Italian Consensus for the Classification and Reporting of Thyroid Cytology 2016; UK RCPath 2016: The UK Royal College of Pathologists System for Reporting Thyroid Cytopathology 2016; RCPA/ASC 2019: The Royal College of Pathologists of Australasia and Australian Society of Cytology 2019; AUS: Atypia of undetermined significance; PPV: Positive Predictive Value; NPV: Negative Predictive Value

Indicators	TBSRTC 2023 [10]	JRSTAC 2019 [6]	ICCRTC 2016 [7]	UK RCPath 2016 [8]	RCPA/ASC 2019 [9]
Quality indicator (AUS percentage)	22.5%	32.5%	20%	27.5%	15%
Quality indicator (AUS/Malignant)	3%	7.5%	4%	5.5%	3%
Diagnostic accuracy	60%	47.5%	52.5%	52.5%	62.5%
PPV of neoplastic cases (4,5,6)	85.71%	100%	72.72%	81.8%	76.47%
NPV of non-neoplastic cases (1&2)	70.58%	66.67%	61.9%	72.2%	70.58%
Sensitivity of neoplastic cases	70.58%	53.8%	50%	64.28%	72.2%
Specificity of neoplastic cases	85.7%	100%	81.25%	86.67%	75%

## Discussion

The present study sought to compare the diagnostic performance of five major thyroid cytology reporting systems: the Japanese system (JRSTAC 2019), Italian system (ICCRTC 2016), Australian system (RCPA/ASC 2019), UK Royal College of Pathologists system (RCPath 2016), and Bethesda system (TBSRTC 2023). The study evaluated several performance indicators for each reporting system, including diagnostic accuracy, positive predictive value (PPV) for neoplastic cases, negative predictive value (NPV) for non-neoplastic cases, sensitivity for detecting neoplastic lesions warranting surgery, specificity for detecting neoplastic lesions warranting surgery, and the following quality indicators, percentage of atypia of undetermined significance (AUS) and AUS/malignant ratio [10].

The diagnostic accuracy across the reporting systems ranged from 47.5% to 62.5%. This highlights that while these reporting systems offer guidance, there is still room for improvement in accurately identifying thyroid lesions. The variation in accuracy could be attributed to differences in categorization criteria, the expertise of pathologists, and the subjective nature of cytological interpretation. The PPV represents the proportion of positive cases in the cytology reports that were confirmed as neoplastic in histopathology. The NPV indicates the proportion of negative cases in the cytology reports that were confirmed as non-neoplastic in histopathology. Higher PPV values indicate that the system is effective in identifying cases that require surgical intervention. The reported PPVs ranged from 72.72% to 100%, suggesting that all reporting systems better identify cases that require surgery. The NPVs ranged from 61.9% to 72.2%, indicating that all reporting systems demonstrated moderate ability to exclude non-neoplastic conditions. The sensitivity values ranged from 50% to 72.2%, and the specificity values ranged from 52% to 85.7%. TBSRTC 2023 and RCPA/ASC 2019 displayed higher overall performance suggesting their effectiveness in correctly identifying neoplastic and non-neoplastic cases.

Higher performance was evident in TBSRTC due to its detailed criterion mentioned per category with subcategorization of AUS by features of cytological and architectural atypia. Similarly, suspicious for papillary thyroid carcinoma listed out different patterns, namely sparsely cellular specimen pattern, incomplete nuclear changes pattern and patchy nuclear changes pattern each with their own set of definitions [16]. These standardized criteria ensure high PPV and also minimize AUS case numbers considerably. Likewise, RCPA/ASC has subcategories in AUS and included background features like psammoma bodies and squamoid cells in addition to nuclear details as isolated criterion for suspicious for malignancy, owing to improved performance [17].

The study's findings underscore the variability in the diagnostic performance of different thyroid cytology reporting systems. TBSRTC 2023 (60%-85.7%) and RCPA/ASC 2019 (62.5%-76.47%) generally demonstrated better overall performance indicators and better quality indicators, suggesting their utility in clinical practice. However, it's important to note that diagnostic accuracy, sensitivity, specificity, PPV, and NPV are influenced by various factors, including the prevalence of disease in the population studied, the experience of pathologists, and the sample size. In addition, physicians need to be vigilant in evaluating reporting systems, factoring in distinct criteria, as variations impact malignant risk and management recommendations given in each reporting system highlighting the importance of nuanced clinical judgement.

Limitations

Several limitations of the study should be acknowledged. The sample size of 40 cases might not be representative of the entire spectrum of thyroid lesions encountered in clinical practice. Additionally, the study focused on comparing reporting systems without accounting for potential interobserver variability between pathologists and radiological correlation. The indeterminate category, Atypia of undetermined significance was not included while assessing performance criteria, as they often require repeat testing. Further research with larger sample sizes, inclusion of all histologic types and involving multiple pathologists could provide more comprehensive insights, especially on indeterminate categories.

## Conclusions

The study's comparison of five widely-used thyroid cytology reporting systems was the first of its kind and provides valuable information about their diagnostic performances. TBSRTC 2023 and RCPA/ASC 2019 appear to exhibit better performance indicators, suggesting their potential effectiveness in clinical practice. However, these findings should be considered alongside the limitations of the study and the broader clinical context.
